# Bessel beam CARS of axially structured samples

**DOI:** 10.1038/srep10991

**Published:** 2015-06-05

**Authors:** Sandro Heuke, Juanjuan Zheng, Denis Akimov, Rainer Heintzmann, Michael Schmitt, Jürgen Popp

**Affiliations:** 1 Leibniz Institute of Photonic Technology (IPHT) Jena e.v., Albert-Einstein-Str. 9, 07745 Jena, Germany; 2 State Key Laboratory of Transient Optics and Photonics,Xi'an Institute of Optics and Precision Mechanics,Chinese Academy of Sciences, Xi'an 710119, P. R. China; 3 Institute of Physical Chemistry and Abbe Center of Photonics,Friedrich-Schiller University Jena, Helmholtzweg 4, 07743 Jena, Germany; 4 King's College London, Randall Division of Cell and Molecular Biophysics, NHH, Guy's Campus, London SE1 1UL, UK

## Abstract

We report about a Bessel beam CARS approach for axial profiling of multi-layer structures. This study presents an experimental implementation for the generation of CARS by Bessel beam excitation using only passive optical elements. Furthermore, an analytical expression is provided describing the generated anti-Stokes field by a homogeneous sample. Based on the concept of coherent transfer functions, the underling resolving power of axially structured geometries is investigated. It is found that through the non-linearity of the CARS process in combination with the folded illumination geometry continuous phase-matching is achieved starting from homogeneous samples up to spatial sample frequencies at twice of the pumping electric field wave. The experimental and analytical findings are modeled by the implementation of the Debye Integral and scalar Green function approach. Finally, the goal of reconstructing an axially layered sample is demonstrated on the basis of the numerically simulated modulus and phase of the anti-Stokes far-field radiation pattern.

Coherent anti-Stokes Raman scattering (CARS) is a versatile tool to investigate the molecular composition of biological and non-biological samples, such as cells or mammalian tissue^1^,^2^. Combining pulsed laser sources with fast laser scanning microscopes (LSM) CARS image acquisition is mostly performed point wise.

As the major competitor to point-wise illumination approachs, a number of wide-field CARS^3^,^4^,^5^,^6^,^7^ configurations were reported receiving information of a multitude of microscopic sample locations while distributing the excitation power over a 3D volume in the range of several micrometers in every direction. Due to the narrow directional distribution of excitation k-vectors offered, the reported configurations require samples with negligible axial extension. Otherwise - if the sample’s k-space representation is not constant in axial direction - phase-matching is achieved only for a limited number of sample K-vectors. Without appropriate correction any resulting image will be unevenly dominated by a few sample frequencies and, thus, represent the sample incompletely. Here, we investigate an alternative focused beam illumination approach that permits the investigation of axially structured samples. Utilizing a Bessel beam as pump to drive the CARS process, an axially extended focal excitation volume is created with cylindrical symmetry along the axial direction. As the major difference to the majority of point wise or wide-field illumination approaches, our Bessel beam approach exploits phase-matching for the characterization of the sample’s micro-structure with the help of an angle-resolved detection.

Non-diffractive Bessel beams were implemented by Durnin almost three decades ago^8^. Ever since, Bessel beams found entrance to various linear microscopy techniques such as optical coherence tomography (OCT)^9^, linear fluorescence microscopy^10^ and recently entered the field of non-linear microscopy, in particular, two-photon excited fluorescence (TPEF)^11^. An extension of this rod-like illumination scheme to CARS microscopy will potentially provide new insights to microbiological processes or speed up the image acquisition time of bulky samples. Compared to TPEF the implementation of Bessel beams into CARS microscopy is complicated due to the requirement for a second excitation source at a shifted wavelength and, more profoundly, by the coherent nature of the CARS process. The present report addresses these issues under the confinement of isotropic, predominantly axial structured samples with negligible non-resonant background. Such requirements are met in reasonable approximation by biological and non-biological samples such as the layered structure of human skin^12^ or for layered dielectric media such as solar cells, respectively.

This paper is organized as follows: first, a basic experimental implementation of Bessel beams for CARS investigations of axially structured samples is presented. Second, a classical description of the generated CARS emission field is provided. Third, the theoretically achievable axial resolution is visualized based on coherent transfer functions. Fourth, numerical results based on the Debye-Wolf integral and scalar Green function are compared to experimental data and the predicted axial resolution capacity is confirmed. As an outlook the possibility of a sample reconstruction from numerically generated far-field radiation data solving the inverse source scattering problem (ISCP) is exemplified.

## Experimental setup

**Figure 1:**
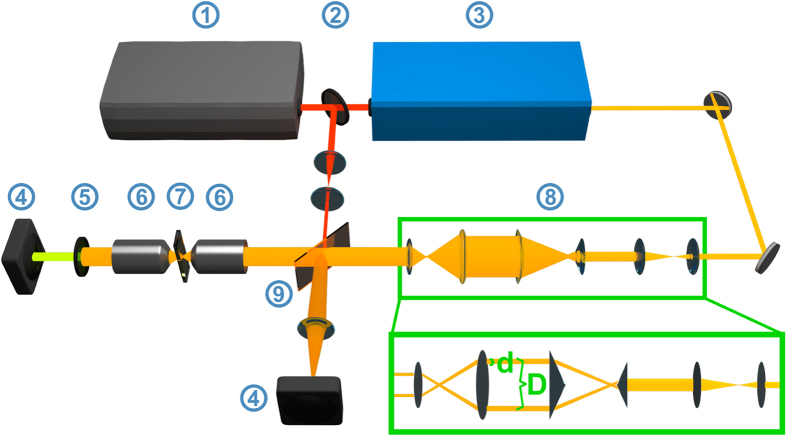
Experimental Setup: Employing the Ti:Sapphire laser directly as Stokes beam its size is reduced to build a weakly focused Gaussian beam after the focusing objective. The pump laser generated by an optic parametric oscillator (OPO) is reshaped by a 6 lenses combination that transforms the incoming Gaussian beam into a narrow (*D*/*d* ≥ 10) collimated laser ring. The insertion in the green box visualizes the ring formation. First, a beam expander increases the beam width. The laser is refracted by the first axicon onto a cone. The second axicon collimates the laser. Preserving the ratio *D*/*d* the beam reducer decreases the ring size to meet the back aperture of the following objective lens. ① Ti:Sa-Laser; ② Beam splitter; ③ Optical parametric oscillator (OPO); ④ Camera; ⑤ Bandpass filter; ⑥ Objective lens; ⑦ Sample; ⑧ Combination of a beam expander, double axicon arrangement and beam size reducer; ⑨ Beam combiner.

The term Bessel beam is ambiguous and refers to non-diffractive beams, whose lateral profile is described over a Bessel function *J*_*n*_ of order n. Here, our considerations shall be restricted to zeroth order Bessel beams. The experimental generation of an ideal Bessel beam is, in general, not possible, since such implementation would require an infinitive amount of energy. Instead a variety of approaches was reported generating beams with reasonable similar properties also referred to as Bessel or Gauss-Bessel beam. Among these experimental implementations axicons^9^, annular apodizing masks^11^ as well as spatial light modulators^13^ are most commonly used in combination with an objective lens to convert Gaussian beams into Gauss-Bessel beams. Depending on the laser source the choice of the converting element might be a critical point, since the efficient generation of CARS requires peak irradiance in the order of GW/cm^2^ at the sample^12^. This becomes more apparent when considering that only about 20% of the incident energy is deposited into the central peak of the Gauss-Bessel beam compared to 85% for an Airy disc as focused by a homogeneous illuminated back aperture of an objective^13^. In addition, the required laser power scales proportionally with the axial extent of the Bessel beam. Therefore, the application of a simple narrow annular apodizing mask inherently losing more than 90% of the incident laser power might be inappropriate if no high peak power laser source (repetition rate ≪ 1 MHz) is available. Though a single axicon features low energy loses its tip causes diffuse scattering. This effect even rises as the Bessel beam axial extension is reduced by decreasing the width of the incident Gaussian beam to obtain sufficient power densities in the focal volume. Lastly, a programmable phase mirror is costly, requires external control and refracts a considerable amount of energy into the unexploited zero order of refraction. As proof of principle experiment, here, a combination of a Keplerian beam expander, two axicons and a Keplerian beam reducer is selected to overcome these limitations of high laser power loses, diffuse scattering and active external control. The effect of this six lenses arrangement is visualized in the green box in fig. 1. The beam expander increases the size of the incident Gaussian beam to reduce the diffuse scattering effect the axicon’s tip. The first axicon refracts the incident beam into a cone of light, while the second axicon collimates the laser to form a narrow ring of light^14^. Note that to obtain a Bessel like lateral profile in the object plane the ratio of the ring diameter *D* and ring width *d* shall be large^15^, i.e., *D*/*d* ≥ 10. The beam reducer adjusts the ring size while preserving the ratio *D*/*d* to fit into the back aperture of the following objective. Directing both colors, i.e., pump and Stokes through the 6 lenses arrangement would result in two Bessel beams which have to be precisely overlapped to allow for an effective elongated focal volume. To avoid such an elaborated alignment procedure the Stokes is employed as a thin Gaussian beam. Passing the objective, the latter is only weakly focused and possesses henceforth a large Rayleigh length and beam waist. Thus, the alignment is simplified. Further technical details concerning the setup such as laser source and selected excitation wavelength are summarized in the supplementary information *- experimental setup*.

Finally, note the similarity of the presented illumination scheme to the concept of USED CARS^16^,^17^. The presented Bessel beam approach can be regarded as a special case of the USED CARS illumination configuration in the limit of a thin pump ring (*D*/*d* ≥ 10) and narrow Stokes Gaussian beam before entering the objectives back aperture. In contrast to USED CARS, however, the Bessel beam approach is intended to maximize the extension of the focal volume while preserving its lateral narrowness to allow for the investigation of z-structured samples rather than to optimize the emission strength of homogeneous samples.

### Classical description

Following Tewari *et al.*^18^ an analytical description for the anti-Stokes radiation can be provided whose theoretical framework is well approximated by the experimental conditions discussed above. At the sample, the pump beam is assumed to be described over a Bessel function of zero order (eq. (1)), while the weakly focused Stokes beam is given in a simplified form by a plane wave (eq. (2)).


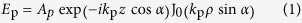



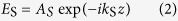


**Figure 2:**
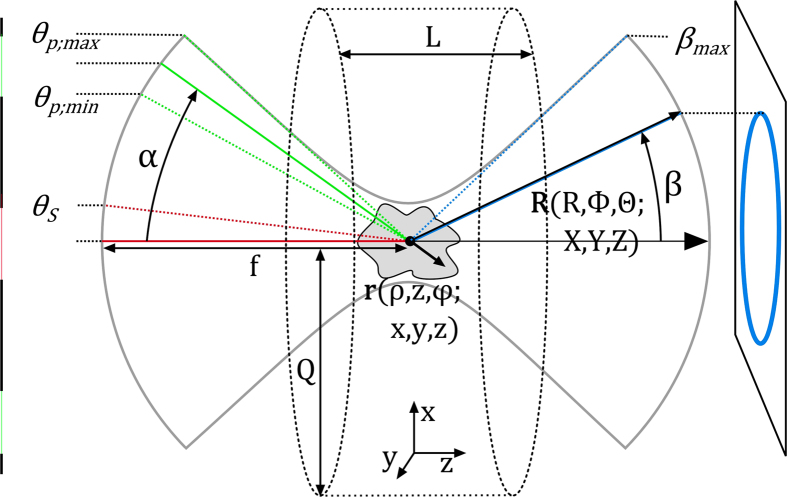
Definition of the parameters used for the calculations. Phase-matching and anti-Stokes radiation angle *β* are controlled by the illumination geometry determined by *θ*_*p;min/max*_ or *α* as well as *θ*_*S*_. Anti-Stokes radiation arises from a homogeneous sample of length L and radius Q or an arbitrary z-structured sample. Pump, Stokes and anti-Stokes beam paths are highlighted in green, red and blue, respectively.

*k*_p_ = 2*πn*/*λ*_p_ and *k*_s_ = 2*πn*/*λ*_s_ are the absolute values of the wave vectors of pump and Stokes beams, respectively. J_0_ is the zeroth order Bessel function, α the angle of radiation incidence (see fig. 2), *z* the preferred propagation direction, *ρ* the radial coordinate and *A*_*p*_, *A*_*s*_ are constant amplitude factors. In the slowly varying envelope approximation (SVEA) the anti-Stokes electrical field generated by isotropic, homogeneously distributed emitters is assumed to be given by the following factorized form (eq. (3)).


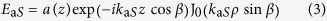


*k*_aS_ is the absolute value of the anti-Stokes wave vector. In cylindrical coordinates the electrical field of the anti-Stokes beam is related to the incident field of the pump and Stokes beam via the inhomogeneous scalar wave equation (eq. (4))^18^,^19^.


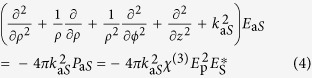


Here, *ϕ* is the angular component completing the cylindrical coordinate system. *χ*^(3)^ is the non-linear susceptibility and *P*_aS_ denotes the third order polarization at the anti-Stokes frequency *ω*_*aS*_. Inserting eqs. (1), (2) and (3) into eq. (4) yields for a homogeneous cylinder-shaped sample of a thickness of *L* and radius *Q*:


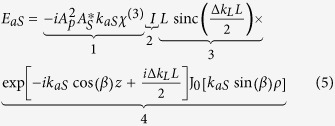


The lateral phase-matching factor *I* is given by


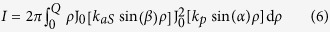


**Figure 3:**
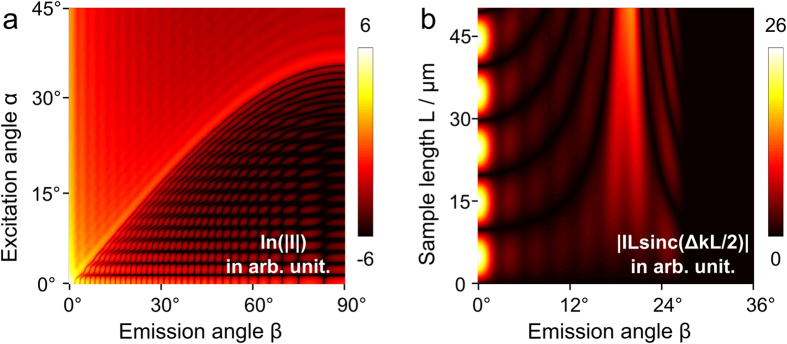
Visualization of the lateral and axial phase-matching factors for *λ*_*p*_ = 671 nm, *λ*_*S*_ = 830 nm and *λ*_*aS*_ = 563 nm. The radius Q in eq. (6) was set to 10 *μ*m, corresponding to a cylindrical sample with a diameter of 20 *μ*m. **a**) Logarithmized absolute value of the lateral phase-matching factor *I* as a function of the incident angle *α* and anti-Stokes emission angle *β*. Due to missing lateral phase-matching almost no anti-Stokes emission is observed for *β* > arcsin[(2*k*_*p*_sin*α*)/*k*_*aS*_]. **b**) Absolute value of the lateral and axial phase-matching factors *IL*sinc(Δ*kL/*2) as a function of the interaction length *L* and anti-Stokes emission angle *β* for the particular incident angle *α* = 15°. Phase-matching will be observed for a homogeneous sample at an anti-Stokes emission angle of approximately 19° at the parameters given above.

Derivation details and the axial phase-matching relation Δ*k*_*L*_ and the definition of the radial scaling factor *M* are provided in the supplementary information *- classical description*. Expression (5) may be separated into 4 factors as outlined. Factor one summarizes constants for given material properties, illumination powers and emission wavelengths. Factor four accounts for the oscillating phase in axial and lateral direction as well as an amplitude modulation due to the zeroth order Bessel function acting as a field multiplier. The third factor represents the axial phase-matching which also appears in the representation of the anti-Stokes field with coaxial propagating pump and the Stokes beam^19^. Less familiar, the second factor describes the lateral phase-matching. The lateral phase-matching factor *I* as well as the combined lateral and axial phase-matching factors *IL*sinc(Δ*k*_*L*_*L/*2) are illustrated as a function of the anti-Stokes emission angle *β* versus the incident angle *α* or the sample thickness *L* in fig. 3, respectively. The lateral phase-matching factor restricts the anti-Stokes emission to a maximum angle *β*_*max*_ that can be readily calculated via 2*k*_*p*_ sin*α* = *k*_*aS*_ sin*β*_*max*_^20^. Independent of the incident angle *α*, anti-Stokes emission is mostly favored in axial direction by the lateral phase-matching factor (see fig. 3). Axial emission, however, will be efficiently suppressed with increasing sample length *L* by the axial phase-matching factor or part 3 of equation (5). Thus, for *L,Q → ∞* the combination of a pump Bessel beam and Stokes plane wave beam results in an anti-Stokes Bessel beam, i.e., a conical beam at anti-Stokes frequency, but with different propagation angle *β*.

### Coherent transfer function - phase-matching for axially micro-structured samples

Provided in the previous section, expression (5) supplies information about strength and direction of the anti-Stokes emission for homogeneous samples. Here, however, structural detail of a non-homogeneous sample shall be determined. A first impression of the obtainable axial resolution of the presented Bessel beam CARS approach can be received via visualization of coherent transfer functions following the considerations in Hashimoto *et al.*^21^ and Hajek *et al.*^22^. Arising from the coherent superposition of the anti-Stokes field, the amplitude at the image plane of an in-focus object is given in scalar approximation as:


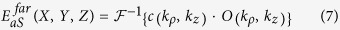


Here, *c*(*k*_*ρ*_, *k*_*z*_) denotes the coherent amplitude transfer function of an objective lens as provided in ref. 23. *X*,*Y* and *Z* represent the Cartesian coordinates of the far-field emission - see also fig. 2. The coherent transfer function depends on the cylindrical coordinates *k*_*ρ*_ and *k*_*z*_ in reciprocal space that correspond to *ρ* and *z* in real space, respectively. 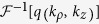 represents the inverse 3D Fourier transform of a function *q*(*k*_*ρ*_, *k*_*z*_), while *O*(*k*_*ρ*_, *k*_*z*_) is the complex emission amplitude in k-space, which can be further decomposed into the local susceptibility and illumination amplitude. Using the convolution theorem the latter is given as:


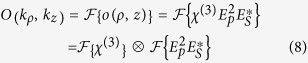


with 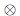 denoting the convolution operation. The susceptibility *χ*^(3)^ can be specified as follows^21^.


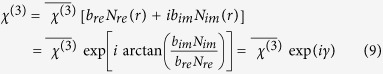

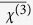
 denotes the average of *χ*^(3)^ while *b*_*re*_ and *b*_*im*_ are its relative real and imaginary part multiplied with the spatially varying density of scatters *N*_*re*_ and *N*_*im*_, which are equal for a negligible non-resonant background. Note, that a non-negligible non-resonant background may be removed by subtraction of the complex amplitudes of the far-field anti-Stokes pattern in- and out-of-resonance. *γ* denotes the average phase shift introduced by an anti-Stokes scattering event. For simplicity, the considerations shall be restricted to strong resonant Raman scatters implying *b*_*re*_*N*_*re*_ ≪ *b*_*im*_*N*_*im*_ with an average phase shift of *π* / 2. Out of resonance, the average phase shift will have to be adapted accordingly.

To account for the influence of the Raman scatter density onto the phase-matching condition for only axially structured samples the Fourier transform of *N*_*im*_ amounts to


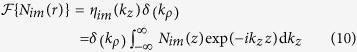


*η*_*im*_(*k*_*z*_) represents the 1D Fourier transform of *N*_*im*_(*z*). Dropping the constants *A*_*p*_ and *A*_*S*_ the illumination amplitude as specified in eqs. (1), (2) and (8) is given in reciprocal space as:


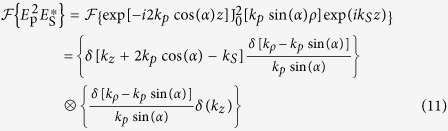


Here, the 3D-Fourier transform is performed in cylindrical coordinates (Hankel transform) using the convolution theorem and the closure relation 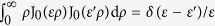
24. Introducing eqs. (8, 9, 10, 11) into eq. (7) leads to


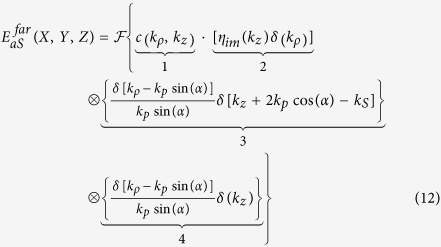


**Figure 4:**
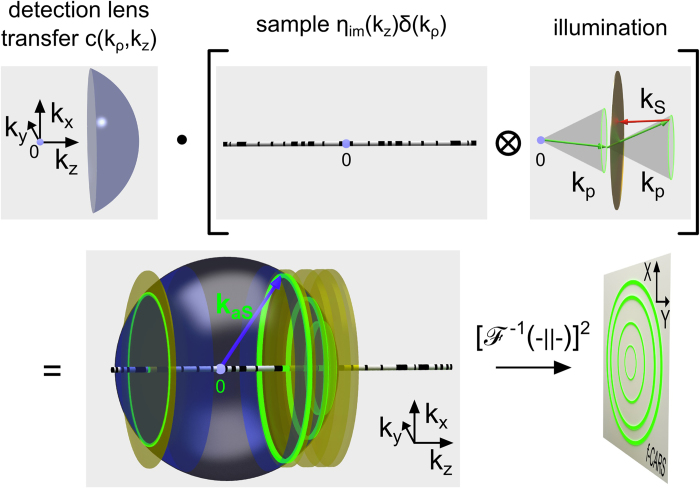
Visualization of the effect of the sample’s micro-structure on the phase-matching condition and emission angle. The arbitrarily z-structured sample displays only contributions on the *k*_*z*_-axis in reciprocal space. Thus, it can be visualized as a rod which is further convolved with the illumination. The latter is represented by a disc shifted in *k*_*z*_-direction by 2cos(*α*)*k*_*p*_−*k*_*S*_. Note that this homogeneous disc is a simplification of the *k*_*ρ*_-profile arising from the 2D convolution of two identical rings of radius sin(*α*)*k*_*p*_ as analytically displayed by factor 4 in equation (12). As a result of the convolution of sample and illumination the complex emission amplitude can be regarded as “brushed” into *k*_*ρ*_-space yielding a stratified structure. The element wise product (Hadamard product) in Fourier space of coherent transfer function with the complex emission amplitude defines which sample Fourier components will observe phase-matching and for which emission angle *β* the corresponding anti-Stokes emission is expected. Consequently, distinct sample Fourier components contribute to the detected anti-Stokes emission in forward and backward direction. A detection of the collimated anti-Stokes emission in forward direction (f-CARS) is symbolized by a detector plane with green rings. Note that for each gray underlaid element the k-space origin is indicated by a small purple sphere in proximity to the number zero.

Equation (12) is visualized in fig. 4. The second part of eq. (12) corresponds to an only axially structured sample and therefore displays only contributions in *k*_*z*_ direction. The sample representation in k-space is convolved with the illumination function (term 3 and 4 of eq. (12)). Simplified, the latter can be illustrated by a thin disc in reciprocal space due to the convolution of pump and probe k-vectors.

As a result of the convolution of sample and illumination (“disc-shaped brush”), the complex emission amplitude is centered on the *k*_*z*_-axis at *k*_*z*_ = 2*k*_*p*_cos(*α*)−*k*_*S*_ + *k*_*z*_ with respect to the sample k-space system and point-wise “brushed” into *k*_*ρ*_-direction. Here, *K*_*z*_ denotes one spatial frequency of the sample *η*_*im*_(*k*_*z*_). Each disc represents one axial spatial frequency of the sample structure. Finally, the complex emission amplitude is cut by a cap of an Ewald sphere, i.e., the representation of the coherent transfer function of the objective lens. Simultaneous phase-matching will be obtained for those emission angles where the discs representing the complex emission amplitude for different sample K-vectors meet the cap of the sphere. Note the similarity of the diagram in fig. 4 with k-space representations obtained for ordinary scattering in the first Born approximation. Note also that the depicted geometry defines a non-linear coordinate transform relating the axial spatial sample frequencies to observed scattering angles. Starting from the origin in k-space all phase-matched *k*_*aS*_ will point towards the intercept of the disc and the spherical cap. Non-surprisingly, different angles of anti-Stokes emission will account for different spatial frequencies of the sample. Yet, tuning the excitation angle and wavelengths, i.e., the proportion of *k*_*p*_ and *k*_*S*_ will not only influence the emission angle for a certain spatial frequency, but will guarantee simultaneous phase-matching of a large variety of the sample’s spatial frequencies for one configuration albeit each at separate detection angles. This property results from the non-linearity of the CARS process - in particular, the convolution of all transverse components of the pump Bessel beam with all transverse components of the probe Bessel beam - see part 4 of eq. (12). It shall be noted that a single combination of sole plane waves of pump, Stokes and probe of any incident angles cannot provide similar information. For a z-structured sample such simple plane wave combination will result in phase-matching and therefore efficient CARS generation only for a single spatial frequency in forward and one in backward direction. A Bessel beam, however, can be regarded as the superposition of a continuum of plane waves with incident angle *α*, which allows for phase-matching of a wide range of sample frequencies.

### Numerical Simulation

Experimental results and the prediction of a high axial resolution implementing Bessel beams for CARS investigations shall be confirmed by numerical calculations. For this purpose, the scalar approximation is dropped in favor of the vectorial Debye theory by Richards and Wolf 25 in combination with scalar Green functions^26^ for two reasons. First, the latter approach also applies for high NA microscope objectives. Second, most recently published numerical calculation studies in nonlinear microscopy base on the combined Debye integral and Green function approach^27^,^28^,^32^. Incorporating the results into an equal theoretical framework will simplify the comparison of the data presented in this study to related work. A summary of the implemented equations is provided in the supplementary information *- numerical calculation methods*.

**Figure 5:**
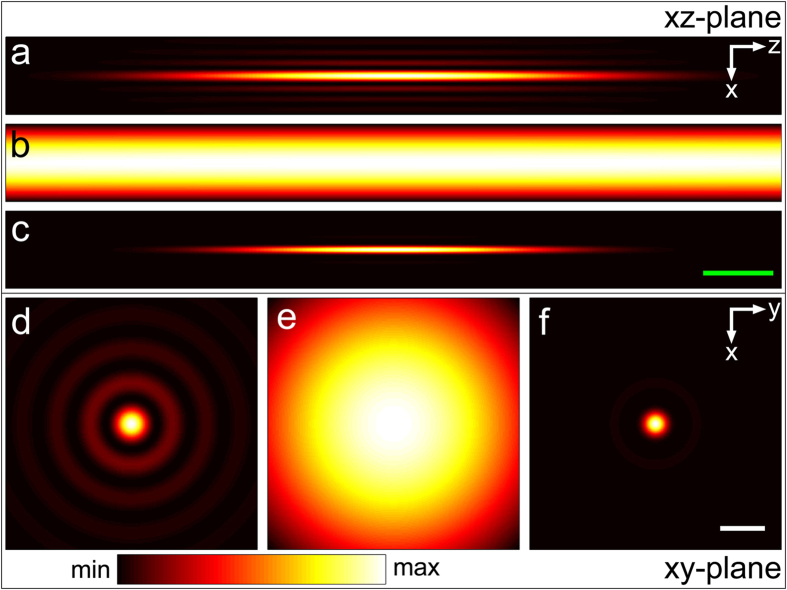
Calculated intensity profile of the Bessel pump (a and d) and Gaussian Stokes (b and e) beam near the nominal focus. For the computation of the Bessel beam the illumination angles *θ*_*p*_ ; *max* and *θ*_*p*_ ; *min* were set to 16° and 13°, respectively. *θ*_*S*_ was set to 4° for the Gaussian Stokes beam. These computational parameters approximately coincide with the experimental parameters used for the generation of image 7(**c**) and 6(**f**). Figure (**c**) and (**f**) present the squared absolute value of the polarization density for a homogeneous sample at anti-Stokes frequency. The polarization density appears rod-like with low contribution of the side lobes which is a desirable effect of the quadratic proportionality between polarization density and electrical field of the Bessel shaped pump beam. The green and white bar in (**c**) and (**f**) equal 10 *μ*m and 1 *μ*m, respectively.

**Figure 6:**
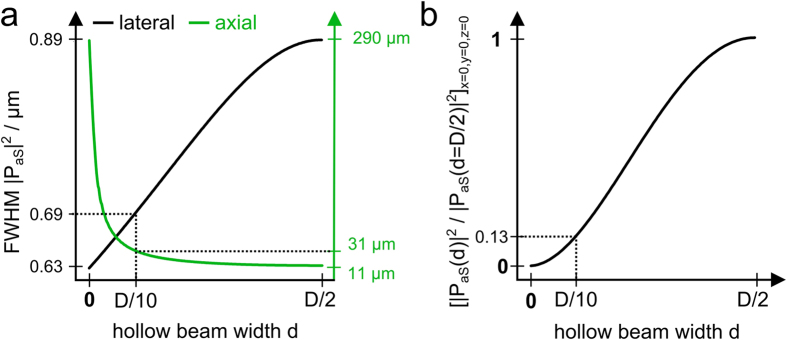
Change of resolution and reduction of the peak squared absolute value of the anti-Stokes polarization as a function of the width *d* of the laser ring. **a**) As the ring width *d* decreases, the lateral resolution increases, while the axial extension of the Bessel beam increases. **b**) With decreasing width *d*, the squared absolute value of the anti-Stokes polarization reduces. The dotted lines in **a**) and **b**) indicate the ratio *D*/*d* that was implemented experimentally. For calculation, the pump and Stokes wavelengths were assumed to be 670 nm and 830 nm, respectively.

The calculations are split into three parts. First, the focal electrical field and polarization density is computed. In a second step the anti-Stokes far-field is calculated at a certain reference plane. Third, the inverse source scattering problem (ISCP) is solved. The result of the first step is displayed in fig. 5 visualizing the intensity of the pump and Stokes beam near the nominal focus. As expected, the annular illumination of the objective’s back aperture results in an elongated focal volume. In contrast, the narrow circular illumination of the objective’s back aperture creates a focus which is both axially and laterally extended. By superimposing the pump and the Stokes beam as indicated in eq. (23) the anti-Stokes polarization density is created. The squared absolute value of the latter for a homogenous sample is presented in fig. 5(c,f). The polarization density can be interpreted as the effective focal volume or the effective source for the observed anti-Stokes emission. As a consequence of the interaction of pump and Stokes field (see eq. (23) the effective focal volume features a shape similar to the pump Bessel beam. Moreover, the quadratic proportionality between the polarization density and Bessel shaped pump field reduces the influence of higher order maxima, which frequently disturb the image formation in linear microscopy using Bessel beams^29^. To visualize the reshaping of the focal excitation field, fig. 6(a) shows the influence of the ratio of ring width d and ring diameter D (see fig. 1) on the lateral resolution as well as the axial extension of the Bessel beam. Furthermore, fig. 6(b) displays the dependence of the squared absolute value of the anti-Stokes polarization at the nominal focus on different ratios of *D/d*.

**Figure 7:**
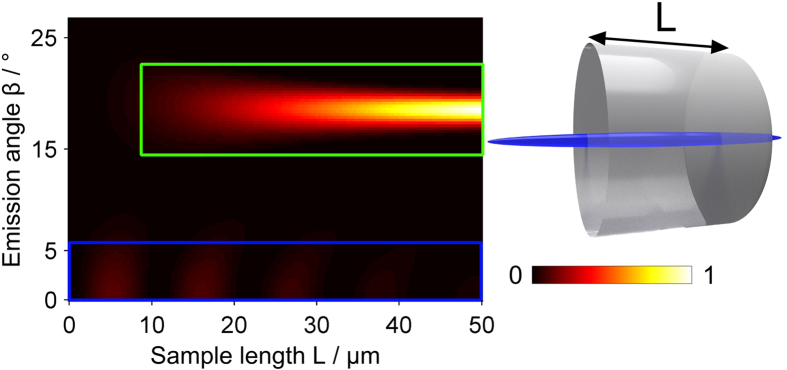
Anti-Stokes intensity of a homogeneous sample of cylindrical shape as a function of the emission angle versus the sample length *L*. As the axial size of the sample increases, the emission strength of the phase-matched contribution grows (highlighted by a green box), while the amount of non-phase-matched emission reduces (outlined by a blue box). For the calculation key parameters were set to: *θ*_*p*;*min*_ = 13°, *θ*_*p*;*max*_ = 16° and *θ*_*S*_ = 4°.

On the basis of the sample’s polarization density the far-field anti-Stokes radiation pattern is computed. Figure 7 presents a plot depicting the influence of the sample thickness *L* on the emission angle for a homogeneous cylindrical sample. Two distinct regions of emission can be identified. The area indicated by a green box is enhanced via 3D phase-matching and therefore increases quadratically in intensity as the sample thickness grows. The second region outlined by the blue box is not supported by 3D phase-matching, but is reinforced by the illumination function, i.e., the convolution of the annular distribution of pump and probe k-vectors favoring low emission angles (see also fig. 3). The axial emission strength displays a sinc^2^(Δ*k*_*L*_*L*/*2*) dependence as predicted by eq. (5). Note that 3D phase-matching requires an axial sample extension that is an order of magnitude larger than the pump wavelength to reinforce non-axial over axial anti-Stokes radiation.

**Figure 8:**
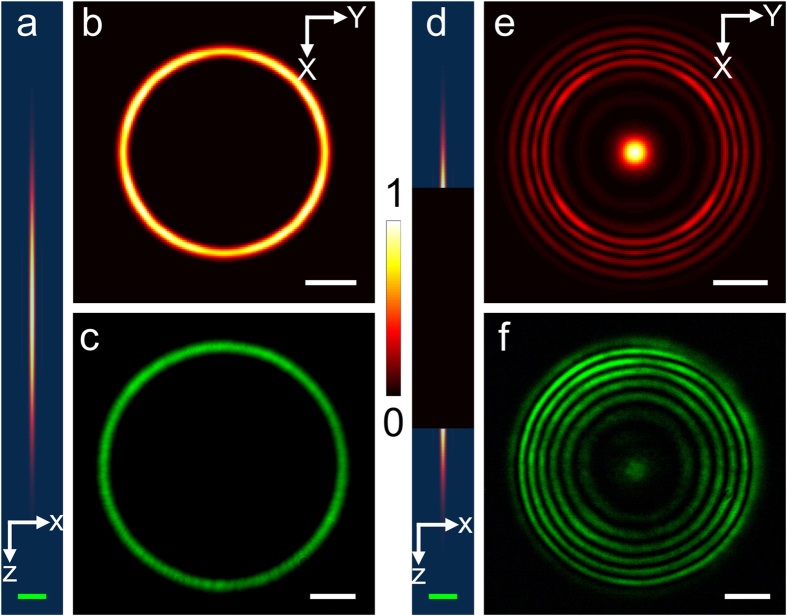
Comparison of numerical and experimental results for a homogeneous sample (a–c) and axially structured sample (d–f). (**a**) and (**d**) show the squared absolute value of the polarization density in zx-plane for a homogeneous sample and a 3 layered sample (Polypropylene (PP); air; PP), respectively. (**b**) and (**e**) display the calculated anti-Stokes intensity plot at a XY detection plane in 5 mm distance of the center of excitation in forward direction. (**c**) and (**f**) highlight the corresponding experimental results. Anti-Stokes radiation of n-octanol is displayed in (**c**). The far-field anti-Stokes radiation pattern of two layers of two z-displaced polypropylene foils with air in between is shown in (**f**). The green and white bar correspond to 4 *μ*m and 1 mm, respectively.

**Figure 9:**
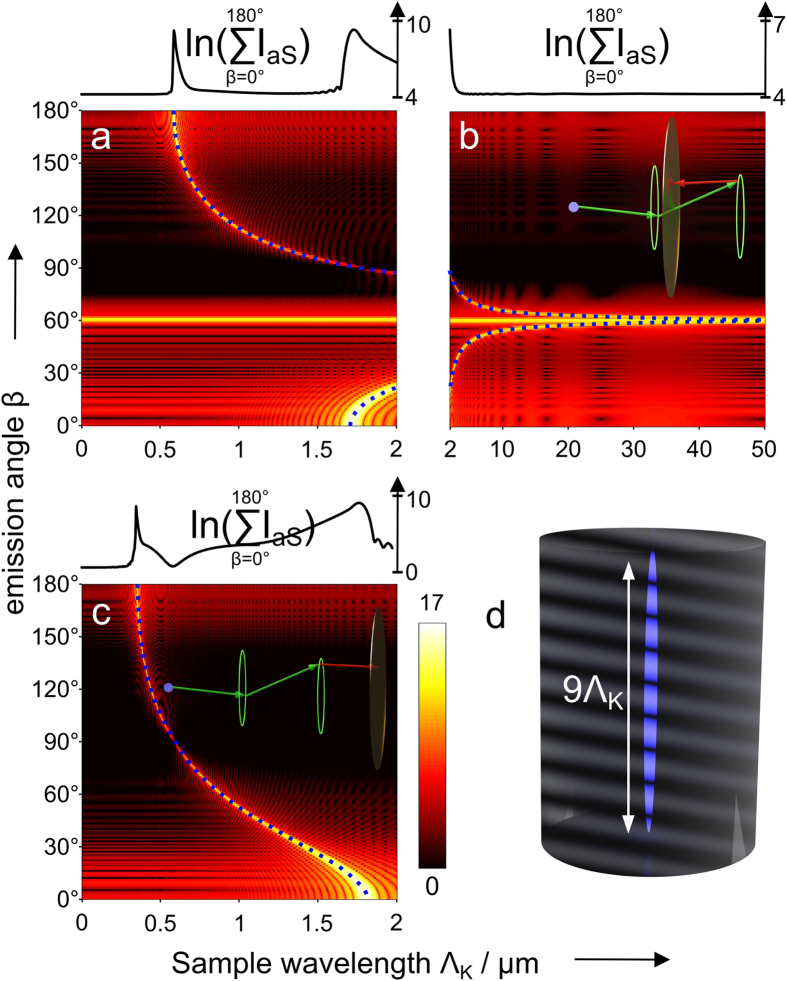
Logarithmic plots of the anti-Stokes intensity as a function of the sample periodicity Λ_*K*_ versus emission angle *β*. The plots and graphs are normalized to the minimum in (**c**). (**a**) and (**b**) Pump (green) and Stokes (red) beam propagate into the same preferred direction. (**c**) The propagation direction of the Stokes beam is inverted. Depending on the sample periodicity distinct anti-Stokes radiation angles are observed. (**d**) Visualization of a periodic sample. The dotted blue line indicate the phase-matching angle(s). The graphs above each plot represents the sum along vertical lines. If anti-Stokes emission is collected in forward and backward direction with *β*_*max*_ ≥ 60°, then two subsequent experiments with condition equal to those assumed for (**a**),(**b**) and (**c**) will provide information about sample z-wavelength beginning from a homogeneous sample down to maximum spatial frequency of 1/585 nm. Calculation parameters: *λ*_*p*_ = 1169.6 nm, *λ*_*S*_ = 1754.4 nm, *λ*_*aS*_ = 877.2 nm, *θ*_*p*;*max*_ = 50°, *θ*_*p*;*min*_ = 48° and *θ*_*S*_ = 4°.

Hence, for an axially extended homogeneous sample the emission pattern is a ring in the XY-plane in forward direction as confirmed experimentally for a cuvette of n-octanol and displayed in fig. 8(c). Note the high emission strength observed for 0° emission angle for the classical description visualized in fig. 3(a). The difference to fig. 8(b,c) arises due to the confined model geometry that sharply truncates the otherwise homogenous polarization density. Opposed to the excitation profile displayed in fig. 5(c), the rectangular polarization density profile of the classic model is characterized by high spatial frequency components K_*z*_ resulting in low emission angles whose emission strength is further enhanced by the illumination, i.e. the convolution of the pump and probe beam. Further, we compared numerically the intensity of anti-Stokes far-field emission under conventional Gaussian beam to the presented Bessel beam illumination for a homogeneous sample in forward direction. For this purpose, the excitation angles for the pump and Stokes beam, i.e. *θ*_*p*_ and *θ*_*S*_ , were assumed to be 13°-16° and 0°-4° for the Bessel illumination, respectively. The excitation angles for the pump and Stokes beam in conventional Gaussian illumination were set to range both from 0-16°. Assuming equal field strength maxima at the nominal focus, the total far-field anti-Stokes intensity of the Gaussian illumination exceeds the Bessel illumination by a factor of 8.5. Note, however, that this factor is a function of the samples microstructure and will vary for non-homogenous samples due to the non-relaxed phase-matching condition for the Bessel illumination - see also fig. 9 and following discussion.

In general, an arbitrary axially structured sample will be characterized by multiple anti-Stokes rings as predicted in the previous section (see 8(e)) and shown in fig. 8(f) for a 3 layered structure of Polypropylene (PP); air; PP. The calculated (fig. 8(b,e)) and corresponding experimentally obtained (fig. 8(c,f) images are in good qualitative agreement. Differences between experimental and calculated images arise from: (1) The neglect of the refractive index variation between sample and air that was set to 1 everywhere for the calculations. (2) The experimental setup that was not appropriately calibrated. (3) The computational model assumes a ring aperture before entering the objective lens, while for the experiment a hollow laser ring is used that was generated by two coaxial axicons without any additional aperture. (4) The sample’s axial micro-structure may not equal exactly the assumed step-index profile, but is characterized by a smoothed transition between polymer layers and air. Thus, high spatial frequencies contribute less to the anti-Stokes far-field pattern compared to the step index profile and, therefore, less emission is observed in fig. 8(f) for lower emission angles. Nevertheless, the qualitative agreement of experiment and calculation mutually corroborate each other and, therefore, build the basis for the pre- and proceeding considerations.

As indicted in eq. (10) every sample may be represented by a superposition of its K-components in Fourier space. Each K_*z*_-component gives rise to a separate phase-matching condition *k*_*aS*_ + *k*_*S*_ = *k*_*pump*_ + *k*_*probe*_ + *K*_*z*_ and hence a ring of anti-Stokes emission with a different emission angle *β*(*K*_*z*_) can be detected.

The finding of different emission angles for different sample frequencies ultimately leads to the question which spatial frequencies may observe phase-matching and further which emission is transferred to a detector. A qualitative access regarding the z-resolution capacity was provided in the previous section. The prediction of continuous phase-matching and radiation collection shall be confirmed here numerically using ideally suited parameters: *α* = 48.2°, *λ*_*S*_ = 1754.4 nm, *λ*_*p*_ = 1169.6 nm, *λ*_*aS*_ = 877.2 nm, Ω = 2850 cm^−1^; detection in forward and backward direction with *β*_*max*_ = 60°. These optimized parameters were obtained by setting the emission angle *β* to 60° for a homogenous sample at 2850 cm^−1^. A figure corresponding to the wavelengths used in the experiment can be found in the supplementary information.

Figure 9 illustrates the dependence of the emission angle *β* for various model samples with periodicity *N*(*z*) = [1 + sin(*K*_*z*_*z*)]/2. The summand 1 of the numerator accounts for a homogeneous scatterer distribution. It provides a reference emission angle in fig. 9(a,b) and guarantees that no non-physical negative concentrations *N*(*z*) or anti-Stokes optical sinks are created. Figure 9(a,b) displays simulation results for which pump and Stokes beam are assumed to propagate in forward direction. Phase-matching for a homogeneous sample will be observed at 60°. The emission angle will decrease until a sample periodicity Λ_*K*_ of about 1.7 *μ*m. Signal in backward direction at 120° arises from the sample periodicity of 0.9 *μ*m and will cut off at 0.585 *μ*m for 180° emission angle. The gap between 1.7 *μ*m and 0.9 *μ*m may be closed by a second experiment where the propagation direction of the Stokes beam is rotated 180°. Consequently, emission from 2*π*/K_*z*_ = 1.7 *μ*m to 0.9 *μ*m will be observed in forward direction for emission angles from 0° to 60°, respectively - see fig. 9(c).

## Discussion and Outlook

**Figure 10:**
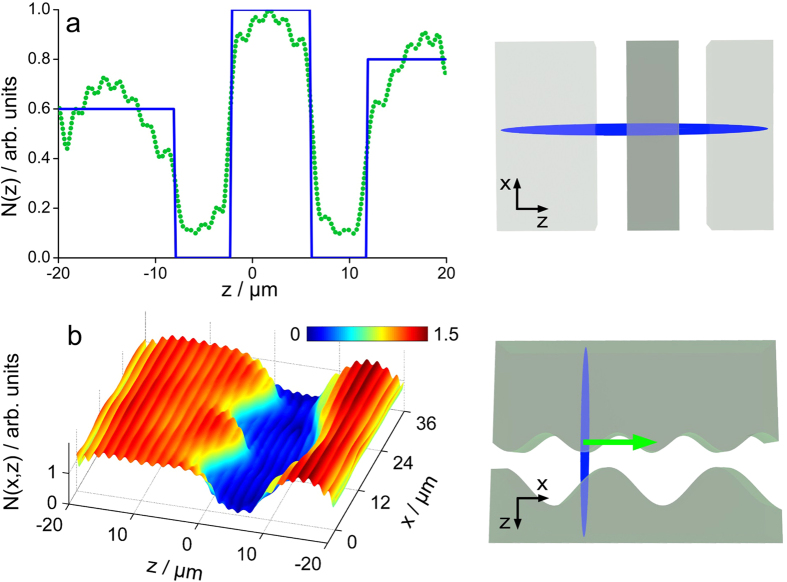
Illustration of the filtered back projection from calculated far-field data. First, the x-polarized anti-Stokes radiation of a z-structured model sample (blue line), e.g. layers of different polymers, was calculated at a reference plane in forward direction. The far-field detection plane covers the anti-Stokes emission angle from *β*_*min*_ 0° to *β*_*min*_ = 60° (NA = 0.87). In a second step the pseudo-inverse of the forward radiation problem is computed and filtered. Multiplication of the pseudo-inverse with the far-field data yields the presented least-square fit of the inverse source scattering problem. The resulting fit is smoothed compared to the true z-profile as a consequence of green function and the limited data set that act as low pass filter. (**a**) Back projected z-structure of an only axially structured sample with varying scatterer concentration along the z-direction. (**b**) xz-plane back projection of a layered sample with smooth variations in x-direction. The green arrow indicates the movement of the polarization density (stretched blue ellipse) into x-direction.

Knowledge about the Fourier components contributing to a sample’s z-structure is just halfway of the ultimate goal of all microscopy: magnified illustration of objects which are beyond the spatial resolution of the naked eye. Thus, means have to be found that translate the information gained from a Bessel CARS experiment into a proper sample reconstruction. Here, the numerically calculated far-field complex amplitude is used to exemplify the reconstruction of a sample’s z-profile. For any particular focus localization the sample is acting as a 3D source. The arising inverse source scattering problem (ISCP) that needs to be solved, however, does not possess a unique solution in general and is ill-posed^30^. Consequently, the solution of the ISCP cannot be identified analytically, but a physical meaningful solution is found by searching for the least-square solution of the ISCP with minimum norm^31^. For this purpose the Moore-Penrose pseudo-inverse of the forward scattering problem was calculated and filtered as outlined in the supplementary information *- filtered backprojection methods*. The filtered back projection from calculated far-field data of a z-structured model sample with varying scatter concentration is displayed in fig. 10(a).

Figure 10(a) proves that a single far-field complex amplitude pattern from a Bessel beam CARS experiment provides sufficient data to reconstruct the z-structured sample’s dept profile. Obviously, the inversion method acts as a low pass filter that eliminates sample frequencies below a certain threshold, in particular, frequencies below 1.7 *μ*m. As indicted previously, complementary experiments - such as a 180° rotation of the Stokes beam direction - will push the frequency limit down to 0.585 nm. Furthermore, the correct z-structure of a sample with smooth variations in *x* and *y* direction can be reconstructed under the computational framework of an only axially structured anti-Stokes radiation sources as displayed in fig. 10(b).

For the back projection from experimental data, information is required about the complete optical geometry, the refractive index of the sample as well as the modulus and phase of the far-field anti-Stokes radiation. The latter may be accessed via phase-retrieval methods such as holography, but was not implemented yet and will be part of our future work.

## Conclusion

A both, experimental and theoretical, study about the utilization of Bessel beams for CARS investigations of mostly axially structured samples was presented. The shown experimental implementation of pump Bessel beams operates without the requirement for high peak power laser sources and, in addition, requires only inexpensive passive optical elements. In particular, other non-linear techniques, such as two-photon excited fluorescence (TPEF), second harmonic generation (SHG) or third harmonic generation (THG) can benefit from adopting our six lens arrangement to laser scanning microscopy, especially, if sufficient excitation power is a critical point. For a homogeneous cylinder-shaped sample, an analytical representation for the anti-Stokes field was derived. Maximum anti-Stokes emission will be observed for large cylinder if both the axial as well as the lateral phase-matching condition are fulfilled. Taking the influence of the sample explicitly into account the phase-matching range of Bessel beams for a z-structured sample was investigated based on coherent transfer functions. The implementation of plane waves only for non-linear scattering microscopy, which is well approximated by e.g. conventional wide-field CARS microscopy, as well as Bessel beams for linear scattering microscopy is not known to provide information about axially structured samples. The combination of both, however, using a plane wave as Stokes and Bessel beam as pump in nonlinear CARS microscopy yields a significant axial resolution. Initially surprising, this effect can be explained via the CARS-specific illumination involving the convolution of pump and probe k-vectors that are located on a cone. Experiments on test samples were performed and the results confirm the numerical calculations based on the Debye integral and scalar Green functions. Assuming optimized experimental parameters in two subsequent experiments, further numerical calculations identified phase-matching and detection of sample periodicity *λ*_*K*_ from 0 to *λ*_*p*_/2. Finally, the possibility to use the detected anti-Stokes emission pattern to perform a sample reconstruction was investigated. Using numerical data the inverse source scattering problem was solved calculating the filtered Moore-Penrose pseudo-inverse of the forward problem in order to least square fit the far-field data and retrieve the sample’s axial micro-structure. Modulus and phase of a single diffraction pattern yields sufficient information to retrieve the depth profile of mostly axially structured samples though weak lateral variations are tolerated, as a key advance over linear coherent techniques. In summary, it is found that Bessel beams bear the potential for fast 1D CARS-profiling that may progress to 3D imaging while scanning the Bessel beam in xy-direction over the sample plane.

## Additional Information

**How to cite this article**: Heuke, S. *et al.* Bessel beam CARS of axially structured samples. *Sci. Rep.* **5**, 10991; doi: 10.1038/srep10991 (2015).

## Supplementary Material

Supplementary Information
