# Supplementary material for: Bessel beam CARS of axially structured samples
Source: Sci Rep. 2015 Jun 5;5:10991. doi: 10.1038/srep10991 (PMC4457012; doi:10.1038/srep10991)
Supplement: Supplementary Information [file srep10991-s1.pdf]

Bessel beam CARS of axially structured samples

Sandro Heuke,[1,2] Juanjuan Zheng,[1,3,2] Denis Akimov,[1,4]
Rainer Heintzmann,[1,4,5] Michael Schmitt,[1,4] and Jürgen Popp[1,4,*]

[1] *Leibniz Institute of Photonic Technology (IPHT) Jena e.v., Albert-Einstein-Str. 9, 07745 Jena, Germany*
[2] *Both authors contributed equally*
[3] *State Key Laboratory of Transient Optics and Photonics, Xi'an Institute of Optics and Precision Mechanics, Chinese Academy of Sciences, Xi'an 710119, P. R. China.*
[4] *Institute of Physical Chemistry and Abbe Center of Photonics, Friedrich-Schiller University Jena, Helmholtzweg 4, 07743 Jena, Germany*
[5] *King's College London, Randall Division of Cell and Molecular Biophysics, NHH, Guy's Campus, London SE1 1UL, U.K.*

[*] Jürgen.Popp@ipht-jena.de

SUPPLEMENTARY INFORMATION

Supplementary information - experimental setup

A schematic of the experimental setup is displayed in figure 1. A continuous wave Neodymium-Vanadate laser with an average power of 18 W operating at 532 nm is used to pump a Coherent Mira HP Titanium-Sapphire laser (Coherent, USA). The Titanium-Sapphire laser generates 2-3 ps pulses (FWHM) with a repetition rate of 76 MHz. The output of the laser at 830 nm is split into two parts. The first part is used directly, i.e., without frequency conversion, as the Stokes beam, the second part is coupled into an optical parametric oscillator (OPO, APE, Berlin). The OPO provides wavelengths continuously variable in the range from 500 to 1600 nm and is used as the pump beam. This allows for tuning the frequency difference of the pump and Stokes pulses at 671 nm to match the CH_2 symmetrical stretching vibration for the CARS measurements. The Stokes beam is directed through a beam reducer (AC254-200-B-ML; Thorlabs AC254-040-B-ML) decreasing the Ti-Sapphire output beam size by a factor of 5. The pump beam is expanded by a Keplerian beam expander (Thorlabs AC254-060-B-ML; Thorlabs AC254-125-B-ML). The extended pump beam is diffracted by a two succeeding axicons (Altechna 1-APX-2-G254, Asphericon X50-200 FPX) to form a collimated laser ring. By changing the distance between the axicons the ring diameter D can be readily adjusted. Finally, the size of the laser ring is adjusted by Keplerian beam size reducer (Edmund Optics 49-390, Thorlabs AC254-030-B-ML) to match the back aperture of the objective. Both beams, i.e. pump and Stokes, are spatially combined by a dichromatic beamsplitter (Semrock FF750-SDi02-25x36) and temporally overlapped using a mechanic delay stage equipped with a retro-reflector. The joined laser beams are coupled into an objective lens (Olympus 10X Plan Fluorite Objective, 0.3 NA) and focused into the sample (cuvette containing n-octanol or a various number of polypropylene layers). The CARS radiation is collected by a combined microscope objective (Olympus 10X Plan Fluorite Objective, 0.3 NA) and achromatic lens (Thorlabs AC254-150-B-ML), frequency filtered (Semrock FF01-650/SP-25 and FF01-563/9-25) and detected by a CMOS camera (Microscopecameras DCM510). A CCD camera (Thorlabs DCC1645C) is used to visualize the approximately 1% reflection of the pump beam by the dichroic filter, which is further focused by a weak lens (Thorlabs AC254-150-B-ML) to monitor the quality of alignment. Sample preparation: 1-octanol (Roth) in a 1 mm cuvette (110-QS, Hellma) was used as a CH_2-rich homogeneous test sample (fig. 8 (c)). The z-structured sample is composed of two layers polypropylene (PP) (Herlitz clear plastic folder) which are glued at two positions. Image 8 (f) was acquired at a position without glue, but with an air filled displacement between the two layers. The displacement was estimated to be 20 μm as confirmed by CARS laser-scanning microscopy. Average at sample was 100 mW (525 W peak power) for the pump Bessel beam and 200 mW (1050 W peak power) for the Stokes Gaussian beam.

Supplementary information - classical description

For convenience eqs. (1)-(4) are repeated:

$$E_{\mathrm{p}} = A_p \exp(-ik_{\mathrm{p}} z \cos\alpha) \mathrm{J}_0(k_{\mathrm{p}} \rho \sin\alpha) \tag{1}$$

$$E_{\mathrm{S}} = A_S \exp(-ik_{\mathrm{S}} z) \tag{2}$$

$$E_{\mathrm{aS}} = a(z) \exp(-ik_{\mathrm{aS}} z \cos\beta) \mathrm{J}_0(k_{\mathrm{aS}} \rho \sin\beta) \tag{3}$$

$$\left(\frac{\partial^2}{\partial\rho^2} + \frac{1}{\rho}\frac{\partial}{\partial\rho} + \frac{1}{\rho^2}\frac{\partial^2}{\partial\phi^2} + \frac{\partial^2}{\partial z^2} + k_{\mathrm{aS}}^2 \right) E_{\mathrm{aS}} = -4\pi k_{\mathrm{aS}}^2 \chi^{(3)} E_{\mathrm{p}}^2 E_{\mathrm{S}}^* \tag{4}$$

Eqs. (1)-(3) are introduced into eq. (4). Neglecting second order derivatives of the amplitude $a(z)$ in slowly varying envelop approximation (SVEA) results eq. (13).

$$\begin{aligned} &2ik_{aS}\cos(\beta)\frac{\partial a(z)}{\partial z}\exp[-ik_{aS}\cos(\beta)z]\mathrm{J}_0[k_{aS}\sin(\beta)\rho] \\ &= 4\pi k_{aS}^2 \chi^{(3)} A_p^2 A_S^* \exp\{-i[2k_p\cos(\alpha) - k_S]z\}\mathrm{J}_0^2[k_p\sin(\alpha)\rho] \end{aligned} \tag{13}$$

Exploiting the cylindrical symmetry of the illumination and sample eq. (13) is multiplied by $\rho \mathrm{J}_0[k_{aS}\sin(\beta)\rho]$ and integrated over ρ from 0 to Q, corresponding to a Hankel transform.

$$\begin{aligned} &2ik_{aS}\cos(\beta)\frac{\partial a(z)}{\partial z}\int_0^Q \rho \mathrm{J}_0^2[k_{aS}\sin(\beta)\rho]\mathrm{d}\rho \\ &= 4\pi k_{aS}^2\chi^{(3)}A_p^2A_S^*\exp\{i[k_{aS}\cos(\beta)-2k_p\cos(\alpha)+k_S]z\}\times\int_0^Q \rho \mathrm{J}_0\left(k_{aS}\rho\sin\beta\right)\mathrm{J}_0^2\left(k_p\rho\sin\alpha\right)\mathrm{d}\rho \end{aligned} \tag{14}$$

The second Lommel integral [24]

$$\int_0^Q \rho \mathrm{J}_0^2(\rho k_{aS}\sin\beta)\mathrm{d}\rho = \frac{Q^2}{2}\left[\mathrm{J}_0^2(Qk_{aS}\sin\beta)+\mathrm{J}_1^2(Qk_{aS}\sin\beta)\right] \tag{15}$$

is used to define the radial scaling factor M as

$$\frac{1}{M} = \cos(\beta)\frac{Q^2}{2}\left[\mathrm{J}_0^2\left(Qk_{aS}\sin\beta\right)+\mathrm{J}_1^2(Qk_{aS}\sin\beta)\right] \tag{16}$$

Introducting M as well as the axial phase-mismatching relation $\Delta k_L = k_{aS}\cos(\beta) - 2k_p\cos(\alpha) + k_S$ into eq. 14 yields

$$\frac{\partial a(z)}{\partial z} = -i2\pi k_{aS}\chi^{(3)}A_p^2A_S^*M\exp(i\Delta k_L z)\times\int_0^Q \rho \mathrm{J}_0[k_{aS}\sin(\beta)\rho]\mathrm{J}_0^2[k_p\sin(\alpha)\rho]\mathrm{d}\rho \tag{17}$$

We define the lateral phase-matching factor as

$$I = 2\pi\int_0^Q \rho \mathrm{J}_0[k_{aS}\sin(\beta)\rho]\mathrm{J}_0^2[k_p\sin(\alpha)\rho]\mathrm{d}\rho \tag{6}$$

and introducing I into eq. (17) returns

$$\frac{\partial a(z)}{\partial z} = -iA_p^2A_S^*k_{aS}\chi^{(3)}MI\exp(i\Delta k_L z) \tag{18}$$

The integration of eq. (18) over z from 0 to L assuming $a(0) = 0$ gives

$$a(L) = -iA_p^2A_S^*k_{aS}\chi^{(3)}MIL\,\mathrm{sinc}\left(\frac{\Delta k_L L}{2}\right)\exp\left[\frac{i\Delta k_L L}{2}\right] \tag{19}$$

Finally, inserting the expression (19) into eq. (3) returns eq. (5).

Supplementary information - numerical calculation methods

All numerical calculations were performed using Matlab (Mathworks).
The angular spectrum representation of a focused field is given by [25, 26]

$$\begin{bmatrix} E_x(\rho,\phi,z) \\ E_y(\rho,\phi,z) \\ E_z(\rho,\phi,z) \end{bmatrix} = \frac{ikf}{2}\exp(-ikf)\begin{bmatrix} I_{00}+I_{02}\cos(2\phi) \\ I_{02}\sin(2\phi) \\ -i2I_{01}\cos(\phi) \end{bmatrix} \tag{20}$$

Where f is the focal length (see fig. 2) of the objective lens and I_{0m} is given by

$$I_{0m} = \int_{\theta_{min}}^{\theta_{max}} E_{inc}(\theta)\sin(\theta)[\cos(\theta)]^{1/2} g_m(\theta) \mathrm{J_m}[k\rho\sin(\theta)]\mathrm{d}\theta \tag{21}$$

Note that θ_{min} signifies our Bessel beam geometry. g_m denotes $1+\cos(\theta)$, $\sin(\theta)$ and $1-\cos(\theta)$ for $m = 0, 1, 2$, respectively. J_m equals the mth order Bessel function and E_{inc}, the incoming electrical field, is provided as:

$$E_{inc}(\theta) = E_0 \exp(-f^2\sin^2(\theta)/\omega_0^2) \tag{22}$$

ω_0 represents the beam waist of a collimated Gaussian beam which is set to 5 mm. The calculations were performed on a grid of 201 voxels in each direction x, y and z with each voxel having a size of 50, 50 and 500 nm, respectively. An expression for the polarization density of the material is generated by the superposition of pump and Stokes beam. The relation between the former and its composing fields is given as:

$$P_{aS,l}^{(3)}(r) = 3\chi_{lmno}^{(3)}(r)E_{p,m}E_{p,n}E_{S,o}^{*} \tag{23}$$

Where l,m,n and o equal x, y or z. Note, that depending on the Raman shift and molecule investigated an additional average phase shift has to be included for proper simulations - see also eq. (9) and following comments. Additionally, the electrical field of pump and Stokes are assumed to be x-polarized in the aperture plane. By applying a Green functions approach the intensity pattern on any screen or camera situated in front of the sample can be computed. The relation between the polarization density and the resulting field is given by

$$\begin{bmatrix} E_{aS,R}(R,\Theta,\Phi) \\ E_{aS,\Theta}(R,\Theta,\Phi) \\ E_{aS,\Phi}(R,\Theta,\Phi) \end{bmatrix} = -\frac{\omega_{aS}^2}{c^2}\frac{\exp(ik_{aS}|R|)}{|R|}\int\int\int_{-\infty}^{\infty}\rho\mathrm{d}\rho\mathrm{d}\phi\mathrm{d}z\frac{\exp(ik_{aS}\mathbf{rR})}{|R|}$$
$$\times\begin{bmatrix} 0 & 0 & 0 \\ \cos(\Theta)\cos(\Phi) & \cos(\Theta)\sin(\Phi) & -\sin(\Theta) \\ -\sin(\Phi) & \cos(\Phi) & 0 \end{bmatrix}\begin{bmatrix} P_{aS,x}^{(3)}(\mathbf{r}) \\ P_{aS,y}^{(3)}(\mathbf{r}) \\ P_{aS,z}^{(3)}(\mathbf{r}) \end{bmatrix} \tag{24}$$

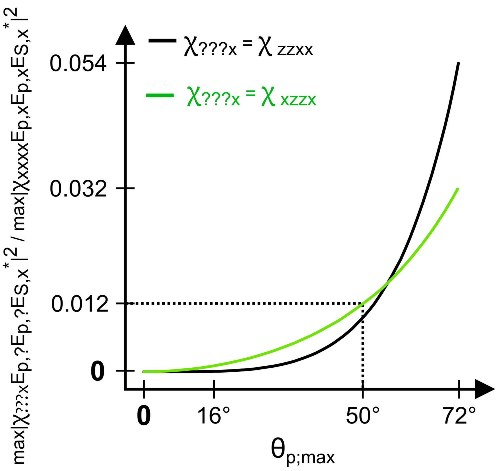

FIG. 11. Contribution of various electric field polarizations near the focal plane. The plot displays the radio of two second largest susceptibility components and the all x-polarized component versus the incident angle. Green curve: $max|\chi_{xzzx}^{(3)}E_{p,z}E_{p,z}E_{S,x}^{*}|^2/max|\chi_{xxxx}^{(3)}E_{p,x}E_{p,x}E_{S,x}^{*}|^2$; Black curve: $max|\chi_{zzxx}^{(3)}E_{p,z}E_{p,x}E_{S,x}^{*}|^2/max|\chi_{xxxx}^{(3)}E_{p,x}E_{p,x}E_{S,x}^{*}|^2$. Calculation parameters: $\chi_{xxxx} = 3\chi_{xzzx} = 3\chi_{zzxx}$; $\theta_{p;max} - \theta_{p;min} = 1°$; $\theta_{S;max} = 4°$; pump wavelength: 671 nm; Stokes wavelength: 830 nm.

It was reported previously that if pump and Stokes beam are x-polarized before entering the objective than contributions from the E_y and E_z can be neglected for CARS microscopy even under tight focusing conditions [32]. This assumption was reevaluated for our Bessel illumination. As evident from fig. 11, the contributions from other

susceptibility tensor component rise with increasing excitation angle α. For highest numerical objective lenses the neglect of E_y and E_z may become inappropriate as a major difference to the conventional point wise illumination [32]. Nevertheless, for the excitation angles used in the experiment and numerical calculation the considerations can be restricted to E_x as a still reasonable approximation. Equation (24) therefore simplifies to eq. (25), which is henceforth implemented for numerical anti-Stokes radiation calculations used for figs. 7-9.

$$\begin{bmatrix} E_{aS,\Theta}(R,\Theta,\Phi) \\ E_{aS,\Phi}(R,\Theta,\Phi) \end{bmatrix} = -\frac{\omega_{aS}^2}{c^2}\frac{\exp(ik_{aS}|R|)}{|R|}\sum_{\rho}^{M}\sum_{\phi}^{N}\sum_{z}^{L}\rho\Delta\rho\Delta\phi\Delta z\frac{\exp(ik_{aS}\mathbf{rR})}{|R|}\begin{bmatrix}\cos(\Theta)\cos(\Phi)P_{aS,x}^{(3)}(\mathbf{r}) \\ -\sin(\Phi)P_{aS,x}^{(3)}(\mathbf{r})\end{bmatrix} \quad (25)$$

Supplementary information - replot of fig. 9 for the wavelengths used in the experiment

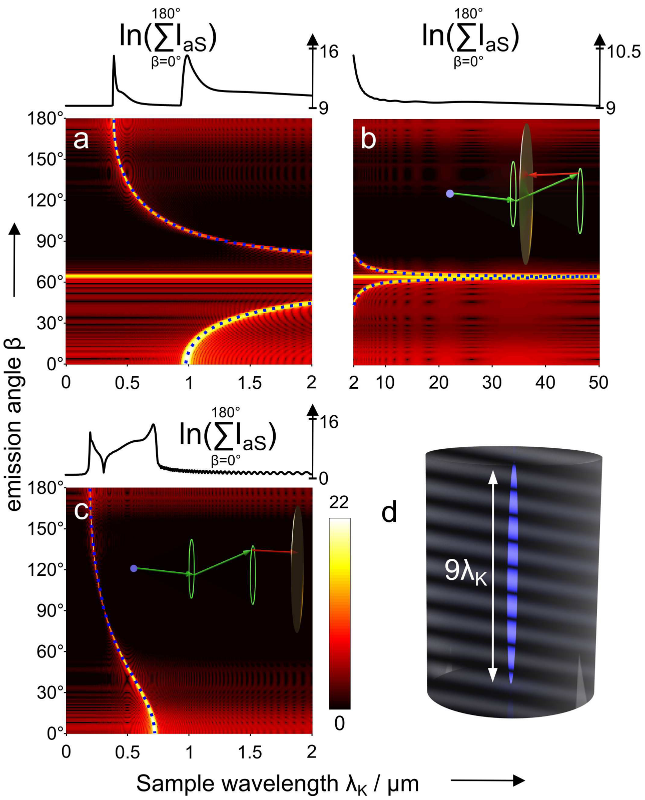

FIG. 12. Logarithmic plots of the anti-Stokes intensity as a function of the sample periodicity Λ_K vs emission angle β. Calculation parameters: $\lambda_p = 671$ nm, $\lambda_S = 830$ nm, $\lambda_{aS} = 563$ nm, $\theta_{p;max} = 50°$, $\theta_{p;min} = 48°$ and $\theta_S = 4°$. For explanation see fig. 9. and following discussion.

Supplementary information - filtered back projection methods

To exemplify the procedure of solving the inverse source scattering problem an anti-Stokes far-field radiation pattern is generated at a detection plane in forward direction of a z-structured model sample. For this purpose the concise formula in eq. (25) could be used, but provides information about the spherical polarized anti-Stokes emission, which is difficult to access experimentally. Much simpler, the x-polarized component may be measured by implementing a linear polarization filter in front of the detector. Thus, the x-polarized far-field anti-Stokes radiation shall be used and can be computed employing a modified version of eq. (24) [26].

$$\begin{bmatrix} E_{aS,X}(\mathbf{R}) \\ E_{aS,Y}(\mathbf{R}) \\ E_{aS,Z}(\mathbf{R}) \end{bmatrix} = -\frac{\omega_{aS}^2}{c^2}\frac{\exp(ik_{aS}|R|)}{|R|}\int\int\int_{-\infty}^{\infty} \mathrm{d}x\mathrm{d}y\mathrm{d}z\frac{\exp(ik_{aS}\mathbf{rR})}{|R|}$$
$$\times \begin{bmatrix} 1-x^2/R^2 & -xy/R^2 & -xz/R^2 \\ -xy/R^2 & 1-y^2/R^2 & -yz/R^2 \\ -xz/R^2 & -yz/R^2 & 1-z^2/R^2 \end{bmatrix} \begin{bmatrix} P_{aS,x}^{(3)}(\mathbf{r}) \\ P_{aS,y}^{(3)}(\mathbf{r}) \\ P_{aS,z}^{(3)}(\mathbf{r}) \end{bmatrix} \tag{26}$$

Using again the relationship $P_{aS,x}^{(3)} \gg P_{aS,y}^{(3)} \gg P_{aS,z}^{(3)}$ the x-polarized far-field anti-Stokes emission can be computed from eq. (27).

$$E_{aS,X}(\mathbf{R}) = \sum_z^L \underbrace{\frac{-\omega_{aS}^2}{c^2}\frac{\exp(ik_{aS}|R|)}{|R|}\sum_x^M\sum_y^N \Delta x\Delta y\Delta z\frac{\exp(ik_{aS}\mathbf{rR})}{|R|}(1-x^2/R^2)3ib_{im}\overline{\chi_{xxxx}^{(3)}}E_{p,x}^2E_{S,x}^*}_{U_z} N(z) \tag{27}$$

Where it was used that $P_{aS,x}^{(3)} = 3\chi_{xxxx}^{(3)}(r)E_{p,x}^2E_{S,x}^* = 3ib_{im}\overline{\chi_{xxxx}^{(3)}}E_{p,x}^2E_{S,x}^*N_{im}(z)$ for a z-structured sample of strong Raman scatters. Equation (27) is rearranged to eq. (28).

$$E_{aS,X}(\mathbf{R}) = \sum_z^L U_z(\mathbf{r},\mathbf{R})N(z)$$

$$\mathbf{E}_{aS,X} = \mathbf{U}\cdot\mathbf{N} \tag{28}$$

Where $\mathbf{E}_{aS,X}$ and $\mathbf{N}$ are column vectors and $\mathbf{U}$ is a rank deficient matrix. For filtering of non-phased-matched high frequency contributions a singular value decomposition (SVD) $\mathbf{U} = \mathbf{M}\ \Sigma\ \mathbf{V}^*$ is performed. Those singular values of Σ are set to zero (truncated singular value decomposition) that are connected to sample frequencies without phase-matching within the numerical aperture of the detection. This regularization procedure allows for back-calculating the z-profile from far-field data that were generated on different grid sizes of the anti-Stokes polarization density. For any measured far-field data the truncation cut-off level of the singular values will have to be adjusted appropriately to account for noise. Finally, using the Moore-Penrose pseudo-inverse algorithm the pseudo-inverse $\mathbf{U}_f^{-1}$ of the filtered $\mathbf{U}_f$ is calculated and multiplied with $\mathbf{E}_{aS,X}$ to obtain the sought-after sample z-profile $\mathbf{N}$.

$$\mathbf{N} = \mathbf{U}_f^{-1}\cdot\mathbf{E}_{aS,X} \tag{29}$$
